# Editorial: Pathogenic microbes: Multi-omics analysis of host-pathogen interactions and immune regulation

**DOI:** 10.3389/fcimb.2022.1053792

**Published:** 2022-10-18

**Authors:** Xuanpeng Wang, Jing Yu, Xin Zhang

**Affiliations:** ^1^ Guangdong Qingyunshan Pharmaceutical Co., Ltd., Shaoguan, China; ^2^ Department of Food Science and Engineering, Ningbo University, Ningbo, China

**Keywords:** intestinal pathogens, host immunity, *yersinia*, *salmonella*, zika virus, group A rotavirus

Intestinal microbiota and enteroviruses can cause gastrointestinal infections in humans, which often have a high incidence of infection, rapid spread, and high incidence of drug resistance. Due to the interactions between the intestinal microbes and host metabolism, gastrointestinal infections may influence the epigenetic regulation and the status of the epigenome of the host. In particular, gastrointestinal infections may be involved in the occurrence of inflammatory bowel disease, autoimmune arthritis, asthma, and other diseases. The aim of this Research Topic is to focus on the use of multi-omics to study the mechanisms of intestinal pathogens and host immunity.

*Yersinia*, commonly known as *Y. pestis*, is the causative bacterium of plague. *Y. pestis* possesses many iron absorption systems. Yersinomycin (Ybt) plays a major role in iron absorption *in vivo* and *in vitro* and in virulence in mice. *FyuA* is a TonB-dependent β-barrel outer membrane receptor (TonB protein) that acts as a receptor for Ybt. Chen et al. constructed mutants of Δ*fyuA* (lack of *fyuA*) and Δ*fyuA*_GCAdel_ (deletion of GCA three bases at positions 915-917 of *fyuA*) and compared them with wild-type (WT) strains. The reduced virulence of the mutant strains in mice may be caused by dysfunctional iron uptake. The Δ*fyuA* and Δ*fyuA*_GCAdel_ strains exhibited lower survival rates in murine RAW264.7 macrophages. In addition, the researchers further explored the transcriptome differences between WT and mutant strains at different temperatures and found that the expression of genes related to Ybt synthesis and its regulation was significantly down-regulated in mutant strains, suggesting that *FyuA* may have a regulatory effect on Ybt. The iron transport system of *Y. pestis* plays an important role in its growth, reproduction, pathogenicity and virulence.

*Salmonella* invasion into the chicken cecum induces transient inflammation mediated by increased gene expression of proinflammatory cytokines and chemokines in intestinal tissue. Chickens develop an inflammatory response, but the signaling pathways that are activated or altered are unknown. Kogut et al. used chicken-specific global immune peptide arrays to investigate changes in cecal immune signaling during the first 24 h after *Salmonella Enteritidis* infection. The findings suggest that colitis increases signaling associated with the innate immune response relative to uninfected control cecum. Acute innate immune signaling is characterized by increased peptide phosphorylation of Toll-like receptor and NOD-like receptor signaling, activation of chemokine signaling, and activation of apoptotic signaling. *Salmonella* infection targets the JAK-STAT pathway and evades the host response by targeting the dephosphorylation of JAK1, TYK2 and STAT1, 2, 3, 4, and 6. The T-cell receptor signaling pathway activates the AP-1 and NF-κB transcription factor cascades. Macrophages are involved in many inflammatory processes in the body and are very important immune cells in the body. Therefore, it is necessary for future studies to explore the polarization of macrophages in the first 24 h after *Salmonella* invades the chicken cecum and whether it mediates related cytokines to regulate the relevant immune signaling pathways in the cecum.

In recent years, the outbreak of Zika virus (ZIKV) around the world has attracted great attention. In endemic areas, the proportion of neonatal microcephaly and adult Guillain-Barre syndrome (GBS) has been significantly increased in the infected population, suggesting that it may have severe neurotoxicity. Shang et al. used two strains of Zika virus with relatively different pathogenicity, the Asian ancestral strain CAM/2010 and the US prevalent strain GZ01/2016, to infect the brains of mice. It was found that both strains elicited strong immune responses. The relatively more pathogenic strain GZ01/2016 elicited stronger immune modulation. The highly pathogenic Zika virus may induce persistent activation of the immune system, resulting in neurological tissue damage. More and more clinical cases and experimental studies have proved that ZIKV is associated with a variety of autoimmune diseases, but its pathogenic mechanism is still unclear. Among them, molecular modeling was considered as the most likely mechanism of action, and this hypothesis was confirmed in ZIKV-triggered GBS. The autoantigen targets that cross-react with ZIKV protein still need further analysis to determine the specific mechanism of ZIKV-induced autoimmune diseases, pathological changes leading to tissue damage, and related protective factors and risk factors need to be further explored. In addition, there are no vaccines or specific drugs that can specifically prevent or treat diseases caused by ZIKV infection, so it is urgent to further clarify the pathogenesis of ZIKV.

Group A rotavirus (RVA) is the most common causative agent of severe acute diarrhea in children under 5 years of age worldwide. Although RVA vaccines can provide homotypic and partial heterotypic protection against several strains, it is necessary to explore genetic and antigenic variation between circulating RVA and vaccine strains. Mao et al. performed a phylogenetic analysis of VP4 and VP7 of RVA strains circulating in China from 2016 to 2019, and identified important antigenic differences that may exist compared with these proteins of vaccine strains to facilitate the adoption of RVA vaccines in China. The VP7 and VP4 sequences of almost all strains share a high degree of homology with previously reported human and vaccine strains. However, in the putative epitopes of VP7 and VP4, multiple amino acid variations were found regardless of the G and P genotypes of these strains. RVA vaccination has significantly reduced the RVA disease burden in children worldwide. Compared with vaccine strains, amino acid differences in epitopes of VP7 and VP4 of Chinese strains may reduce the effectiveness of vaccines, and further study of these epitopes is required. In addition, efforts to accelerate the development of new RVA vaccines, including reverse genetics, are warranted.

In the future, research combining omics-data with intestinal host-pathogen interactions and linking their functions to help us better understand their specific roles in the immune mechanisms of intestinal infectious diseases, and host-pathogen interactions with specific molecular mechanisms in human health and disease.

## Author contributions

Conceptualization, writing—original draft, XW; writing—review and editing, JY; supervision, writing—original draft, XZ. All authors contributed to the article and approved the submitted version.

## Funding

This work was sponsored by the Ningbo Natural Science Foundation (2021J107).

## Conflict of interest

Authors XW and JY were employed by Guangdong Qingyunshan Pharmaceutical Co., Ltd.

The remaining authors declare that the research was conducted in the absence of any commercial or financial relationships that could be construed as a potential conflict of interest.

## Publisher’s note

All claims expressed in this article are solely those of the authors and do not necessarily represent those of their affiliated organizations, or those of the publisher, the editors and the reviewers. Any product that may be evaluated in this article, or claim that may be made by its manufacturer, is not guaranteed or endorsed by the publisher.

